# Fire safety in the eye theatre

**Published:** 2013

**Authors:** Brian R Savage

**Affiliations:** Ophthalmologist, Mvumi Hospital, Dodoma, Tanzania

**Figure:**
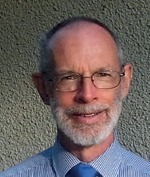
Brian R Savage

Recently while doing cataract surgery, I saw a visiting student wandering across our theatre unaware that he was trailing flames from his gown. Deserting my patient, I beat on the back of the unsuspecting student with my surgically gloved hands (!) to put out the flames. The student was not harmed except for a hole in his gown, though my hands were a bit scorched, and the seeds of this article had been sown.

Fire in the eye theatre is, fortunately, a rare occurrence. Should there be an outbreak of fire, however, there is serious risk of injury to patients, who because of blindness and age may be less able to make a quick escape, also to staff and students, especially at times of greater risk (e.g. if the theatre is busy and crowded).

The three theatre fires of my experience have all been due to the use of a methylated spirit lamp as a low-cost means of heating cautery. Although economical, the method has a number of disadvantages:

- The 70percnt; aqueous spirit usually available is difficult to ignite, consuming time and many matches.
- Therefore once burning, the naked flame may be left burning for the duration of a list.
- If the burning lamp is placed near a window, the burning flame may not be visible in the light
- If placed (as is common) on a low stool in the centre of the theatre, near to the instrument nurse, the lamp is just at the right height to ignite a low-hanging drape, or the folds of the operating gown, or clothes, of staff or students who are passing by, or who are leaning over, engrossed in the progress of an operation.

Spirit lamp fires are easily preventable:

- If possible, do not use them in the first place. Use an alternative method which can be switched on and off easily, notably a cheap cigarette lighter,^1^ or a battery-driven or mains powered electrical cautery machine. Electrical cautery however tends to be more expensive, and require special precautions to make sure of sterility.
- If for some reason there is no alternative, then the lamp must only be ignited at the precise time when required, by an assistant for whom this is the sole responsibility, and the flame should be extinguished **as soon as** cautery has been performed.

**Figure:**
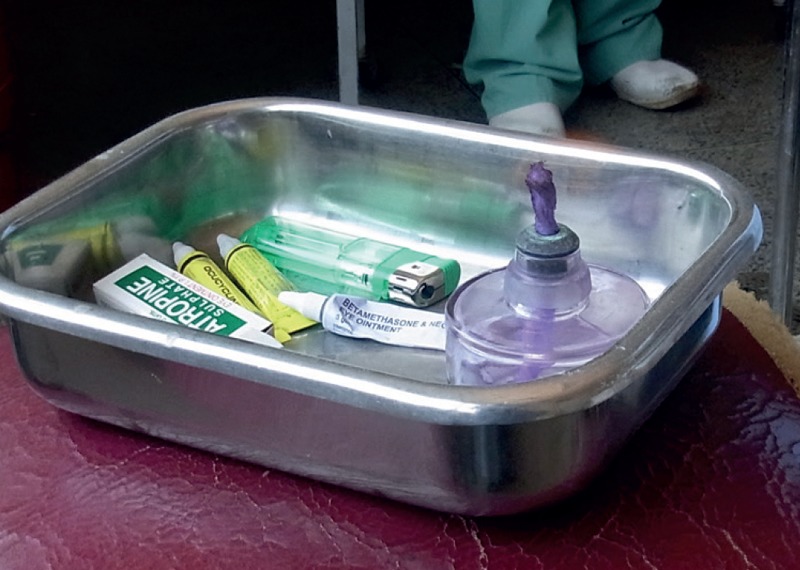
This spirit lamp is alight, but the flame is hardly visible in the bright light, coming from a window nearby.

**Figure:**
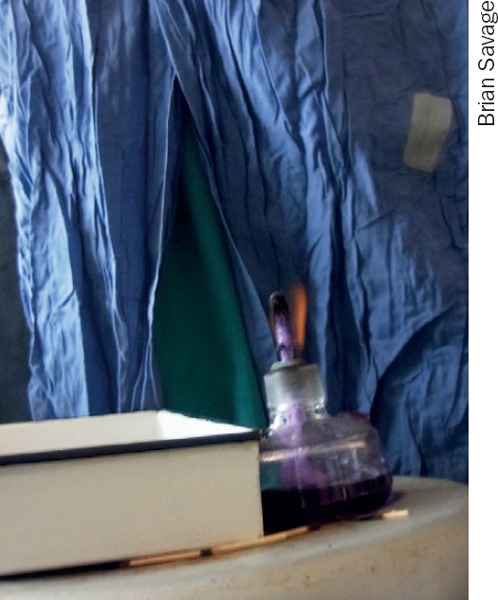
Lighted spirit lamp on a low stool very close to the gown of a scrub nurse attentive only to the operation.

**Figure:**
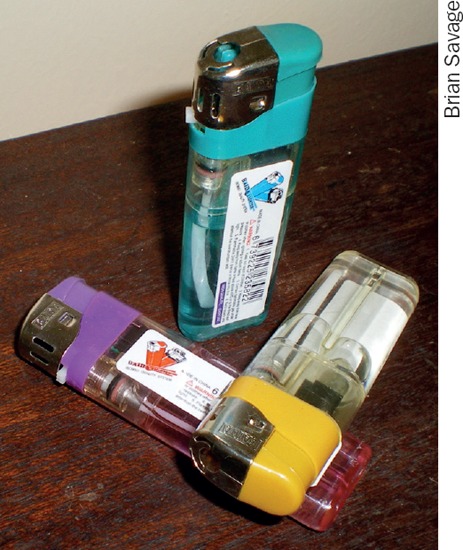
Inexpensive cigarette lighters of a type available locally can be ignited and extinguished easily and safely.

**Figure:**
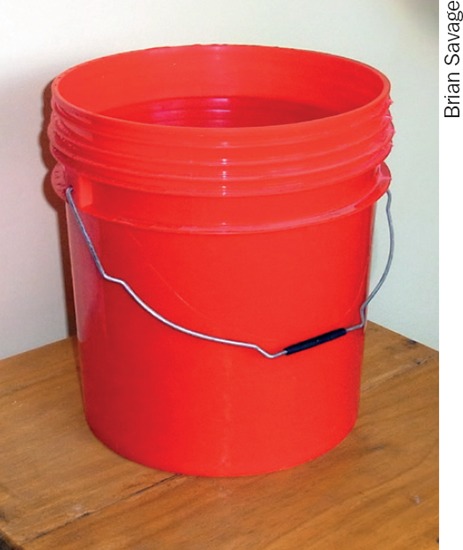
Any bucket will do as long as it can be easily lifted by any member of staff.

**Figure:**
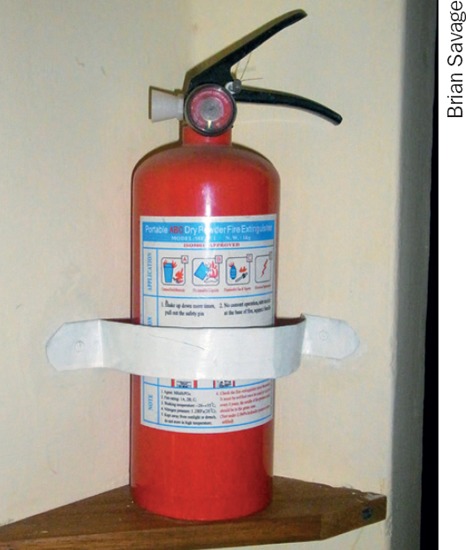
Dry powder fire extinguisher. Note the white pointer on the dial is in the green (safe) range. Check it annually with the fire department.

Other sources of theatre fires include:

- electrical faults
- fuel such as gas or kerosene for stoves used in instrument sterilisation, or petrol used in a generator.
- use of a naked flame in the vicinity of gaseous oxygen used for anaesthetic purposes.
- spent matches thrown inadvertently into waste material while still alight.

Prevention is not difficult, and I suggest the following:

1. Have a meeting with staff to inform them about, and discuss with them, the dangers of fire in the theatre.
2. Draw up (jointly) a set of simple rules for preventing fire, and also what to do if a fire starts.
3. Mark emergency exits in the theatre, and make sure they can be easily opened if there is a fire. If they are locked, the **key must be available in the door**.
4. Have a bucket of water and/or sand and fire extinguishers present in the theatre. Make sure staff know how to operate fire extinguishers and have the extinguishers checked and refilled annually or after use.
5. Do not have a naked flame in the presence of anaesthetic oxygen.
6. Keep fuels such as gas, kerosene or petrol, and equipment using these fuels, outside the theatre. They should be used in a well-ventilated room with an escape route in case of fire.
7. **Always** turn off stoves or generators **before** refilling, **never** refill while they are still running.
8. Know where electrical appliances and mains power can be switched off in case of an electrical fire or fault.
9. Get an electrician to check the safety of the theatre electricity supply.

Finally it is worth examining the hospital insurance policy to see whether harm to patients, staff and equipment is covered if a fire took place in your eye theatre.
